# Sequential Add-On Therapy Modifies Mortality Risk Stratification in Group 1.4 Pulmonary Arterial Hypertension: A Real-World, Single-Center Retrospective Cohort Study from Mexico

**DOI:** 10.3390/jcm15134924

**Published:** 2026-06-24

**Authors:** Arturo Cortes-Telles, Yuliana Valeria Priego-Escamilla, Diana Lizbeth Ortíz-Farias, Saúl Vázquez-López, Yuri Noemí Pou-Aguilar, Esperanza Figueroa-Hurtado

**Affiliations:** Clínica de Enfermedades Respiratorias del Hospital Regional de Alta Especialidad de la Península de Yucatán, IMSS-Bienestar, Mérida 97130, Mexico; yulipriegoe@gmail.com (Y.V.P.-E.); dianalof16@gmail.com (D.L.O.-F.); saul.vazquez2@hotmail.com (S.V.-L.); yunopo@hotmail.com (Y.N.P.-A.); esperfh@hotmail.com (E.F.-H.)

**Keywords:** sequential add-on therapy, COMPERA 2.0, Eisenmenger syndrome, pulmonary arterial hypertension, REVEAL Lite 2

## Abstract

**Background:** Dynamic risk stratification is fundamental to the modern management of pulmonary arterial hypertension (PAH). However, data on the impact of sequential add-on therapy in patients with Group 1.4 PAH—particularly in Latin American populations—remains limited. This study evaluated changes in risk classification using COMPERA 2.0 and REVEAL Lite 2 scores in patients treated with endothelin receptor antagonist (ERA) and phosphodiesterase type 5 inhibitor (PDE5i) combination therapy (macitentan + sildenafil) at a referral center in Mexico. **Methods:** In this single-center, retrospective cohort study, 25 patients with a confirmed diagnosis of PAH between 1st January 2022 and 31st December 2024 were evaluated at baseline and after 24 weeks of treatment. Clinical, functional, and biochemical parameters were recorded. Within-patient changes were analyzed using the Wilcoxon signed-rank test, and agreement between risk assessment tools was assessed using Spearman’s correlation coefficient. **Results:** At 24 weeks, patients demonstrated significant improvement in World Health Organization functional class (*p* = 0.002) and a significant reduction in brain natriuretic peptide levels (*p* = 0.003). Both COMPERA 2.0 and REVEAL Lite 2 scores showed a consistent shift toward lower-risk categories. A strong concordance between the two tools was observed. **Conclusions:** Sequential add-on ERA + PDE5i therapy was associated with meaningful improvement in risk stratification among patients with Group 1.4 PAH. These findings support the clinical utility of simplified, noninvasive risk assessment tools in real-world settings, particularly in resource-constrained environments.

## 1. Introduction

Pulmonary arterial hypertension (PAH) is a progressive and life-threatening disorder characterized by pulmonary vascular remodeling, sustained elevation of pulmonary vascular resistance, and chronic right ventricular overload, ultimately leading to right heart failure and premature death. Despite advances in targeted therapies over the past decade, PAH continues to be associated with substantial morbidity, high mortality rates, and significant impairment in quality of life [1]. Epidemiological estimates indicate an annual incidence of one to two cases per million and a prevalence ranging from 15 to 50 cases per million inhabitants. More recent data suggest a shift toward older age at diagnosis and an increasing burden of cardiovascular comorbidities [2,3].

Within the PAH classification, Group 1.4 represents a distinct and relatively understudied clinical phenotype. It is characterized by multisystem involvement, progressive right ventricular dysfunction, and unfavorable clinical outcomes. Compared with idiopathic PAH, evidence guiding therapeutic strategies in this subgroup remains limited, highlighting an important gap in the literature [4,5].

Contemporary management of PAH is centered on dynamic risk stratification. The 2022 European Society of Cardiology/European Respiratory Society (ESC/ERS) guidelines recommend a multiparametric approach that categorizes patients into four risk strata—low, intermediate–low, intermediate–high, and high—based on clinical, functional, and biochemical parameters. This framework informs treatment decisions and aims to optimize long-term outcomes [1]. Importantly, achieving and maintaining a low-risk profile during follow-up has been consistently associated with improved survival, underscoring the need for regular reassessment beyond the time of diagnosis [6]. The Comparative, Prospective Registry of Newly Initiated Therapies for Pulmonary Hypertension (COMPERA 2.0) model refines this strategy by offering a simplified and reproducible tool suitable for routine clinical practice [7].

In parallel, the Registry to Evaluate Early and Long-term PAH Disease Management Lite 2 (REVEAL Lite 2) score was developed as a streamlined version of the REVEAL 2.0 model. It incorporates six noninvasive variables—functional class, systolic blood pressure, heart rate, 6 min walk distance (6MWD), brain natriuretic peptide (BNP) or N-terminal pro B-type natriuretic peptide (NT-proBNP) levels, and renal function—while preserving adequate predictive performance for 1 yr mortality. Among these variables, natriuretic peptides have demonstrated the strongest prognostic value, reinforcing their role as dynamic biomarkers of disease severity and treatment response [8].

There is growing evidence supporting the early use of combination therapy targeting multiple pathogenic pathways in PAH. The combination of endothelin receptor antagonists (ERAs) and phosphodiesterase type 5 inhibitors (PDE5i) exerts complementary effects on the endothelin and nitric oxide pathways, resulting in improved hemodynamic and clinical outcomes. The AMBITION trial demonstrated that initial combination therapy with ambrisentan and tadalafil significantly reduced the risk of clinical failure compared with monotherapy in treatment-naïve patients, primarily by decreasing PAH-related hospitalizations [9]. These findings have reinforced current guideline recommendations favoring sequential add-on therapy in most patients with PAH [1,9].

Nevertheless, real-world data indicate that the implementation of guideline-recommended therapies remains inconsistent. A substantial proportion of patients—particularly those in intermediate- or high-risk categories—continue to receive monotherapy [10]. In Latin America, additional barriers include limited access to specialized care, inconsistent use of standardized risk assessment tools, and restricted availability of therapies [11,12]. In Mexico, data specifically evaluating risk stratification in the context of sequential add-on therapy are scarce, reflecting a persistent gap between evidence-based recommendations and clinical practice [13,14].

Accordingly, the present study aimed to evaluate changes in risk stratification, as assessed by COMPERA 2.0 and REVEAL Lite 2 scores, following the addition of ERA to PDE5i as sequential add-on therapy over 24 weeks in patients with Group 1.4 PAH.

## 2. Materials and Methods

### 2.1. Study Design and Ethical Approval

This single-center retrospective cohort study included adult patients with a confirmed diagnosis of PAH between 1 January 2022 and 31 December 2024. All patients were classified as Group 1.4 according to the 2022 ESC/ERS guidelines [1] and were followed at the Respiratory Diseases Clinic of the Hospital Regional de Alta Especialidad de la Península de Yucatán—IMSS Bienestar.

The study protocol was approved by the Institutional Research and Ethics Committee (CONBIOETICA No. 31-CEI-001-231011; Registration No. 2023-008) and conducted in accordance with the Declaration of Helsinki. To ensure data confidentiality, all patient information was anonymized and stored on a password-protected computer accessible only to the principal investigator.

The study adhered to the Strengthening the Reporting of Observational Studies in Epidemiology (STROBE) guidelines [15]. Clinical data were systematically extracted from the medical records of patients under active follow-up. The diagnosis of PAH was confirmed by right heart catheterization, in accordance with the ESC/ERS 2022 criteria [1]. Because of the limited number of eligible patients, a convenience sampling approach was adopted, including all individuals who met the eligibility criteria and had at least one follow-up evaluation after initiation of combination therapy.

### 2.2. Participants

Adult patients (≥18 yr) with a confirmed diagnosis of Group 1.4 PAH were eligible for inclusion. All patients had previously received monotherapy (sildenafil) and were under regular follow-up at the specialized clinic. Baseline was defined as the time when all patients initiated sequential add-on therapy with an ERA (macitentan) plus PDE5i (sildenafil), and follow-up assessments were conducted at 24 weeks.

All patients had complete clinical records at both time points. Only patients with PAH classified as Groups 2, 3, 4, or 5, or Group 1 subtypes other than 1.4, were excluded. Additional exclusion criteria included treatment with intravenous or subcutaneous prostacyclin analogs at baseline and any physical limitation that precluded completion of the 6MWD test.

### 2.3. Clinical, Biochemical, and Imaging Variables

Clinical, functional, biochemical, and imaging data were systematically collected from medical records. Clinical variables included dyspnea severity assessed using the modified Medical Research Council (mMRC) scale and functional status according to the World Health Organization (WHO) classification.

Functional capacity was evaluated using the 6MWD test. Biochemical variables included serum levels of BNP and creatinine. Imaging parameters were obtained from transthoracic echocardiography (TTE) and included systolic pulmonary artery pressure, tricuspid annular plane systolic excursion (TAPSE), and tricuspid regurgitation jet velocity.

### 2.4. Risk Stratification

Risk stratification was performed using two validated tools for PAH: COMPERA 2.0 and REVEAL Lite 2. Assessments were conducted at baseline and at 24 weeks following initiation of combination therapy.

Patients were categorized according to established thresholds. For COMPERA 2.0, patients were classified as low, intermediate–low, intermediate–high, or high risk. For REVEAL Lite 2, patients were stratified as low risk (score 1–5), intermediate risk (score 6–7), or high risk (score ≥ 8) [7,8].

### 2.5. Statistical Analysis

Descriptive statistics were used to summarize baseline characteristics and follow-up data. Continuous variables are presented as appropriate measures of central tendency and dispersion, while categorical variables are expressed as frequencies and percentages.

Given the non-normal distribution of several variables and the ordinal nature of risk categories, within-subject changes between baseline and 24 weeks were analyzed using the Wilcoxon signed-rank test.

Agreement between COMPERA 2.0 and REVEAL Lite 2 risk classifications was assessed using Spearman’s rank correlation coefficient (*ρ*). To explore whether longitudinal changes differed according to the presence of Eisenmenger syndrome, a mixed analysis of variance was performed. Because several variables deviated from normality, we employed the Aligned Rank Transform (ART) procedure.

Due to the high variability in BNP levels, additional analyses were conducted to evaluate temporal changes. Although BNP values were log-transformed for graphical representation, all statistical analyses were performed using the original (non-transformed) data.

All statistical analyses were conducted using JAMOVI software (The JAMOVI project, 2025, version 2.7.6. Sydney, Australia). The ART analyses were conducted using the ARTool package in R (Northwestern University, Washington, DC, USA) and graphical visualizations were generated in R using the *ggplot2* package (Springer-Verlag, New York, NY, USA).

## 3. Results

A total of 25 patients were included in the analysis. Baseline demographic and clinical characteristics are summarized in Table 1. The median age was 37 yr (interquartile range [IQR]: 31–49), and the cohort was predominantly female (96%). The median body mass index (BMI) was 28.1 kg/m^2^ (IQR: 25.8–31.1).

Regarding PAH classification, most patients (72%, *n* = 18) had PAH associated with congenital heart disease (PAH-CHD, Group 1.4.4), whereas 24% (*n* = 7) had PAH associated with connective tissue disease (PAH-CTD). Overall, 64% of patients were diagnosed with Eisenmenger syndrome.

### 3.1. Changes in Clinical, Functional, and Echocardiographic Variables

**Figure 1:**
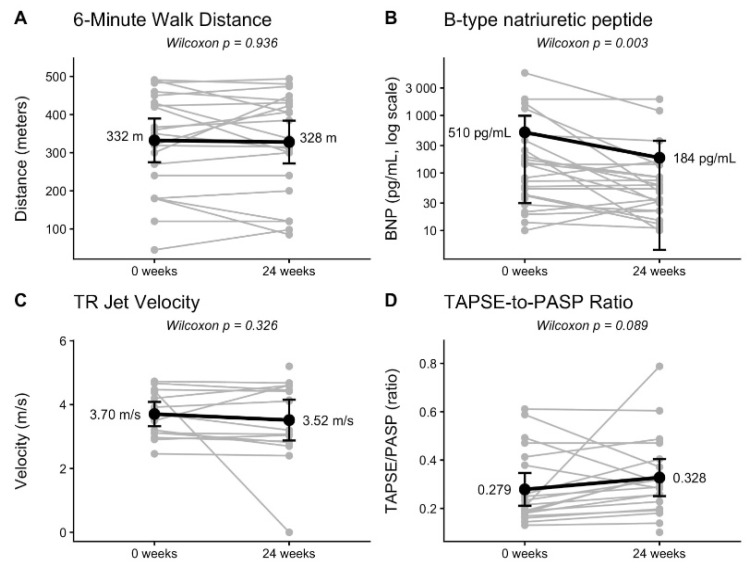
Individual and mean changes in clinical variables at baseline (0 weeks) and at 24 weeks: (**A**) 6 min walk distance (6MWD); (**B**) brain natriuretic peptide (BNP) levels, presented on a logarithmic scale; (**C**) tricuspid regurgitation jet velocity; and (**D**) TAPSE/sPAP ratio. *p* values correspond to the Wilcoxon signed-rank test.

Most clinical and functional parameters remained stable over the 24-week follow-up period (Figure 1). The 6MWD showed no significant change (332 vs. 328 m; *p* = 0.936; Figure 1A), nor did tricuspid regurgitation jet velocity (3.70 vs. 3.52 m/s; *p* = 0.326; Figure 1C).

The TAPSE/PASP ratio demonstrated a trend toward improvement (0.279 vs. 0.328), although this did not reach statistical significance (*p* = 0.089; Figure 1D). In contrast, BNP levels decreased significantly from baseline to 24 weeks (510 vs. 184 pg/mL; *p* = 0.003; Figure 1B).

**Figure 2:**
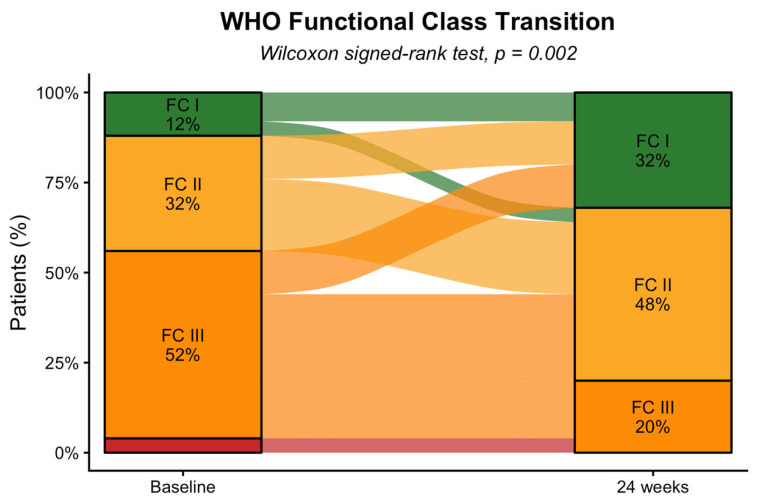
Changes in World Health Organization (WHO) functional class from baseline to 24 weeks. The width of the bands represents the proportion of patients transitioning between categories. A trend toward less severe functional classes was observed during follow-up. *p* values correspond to the Wilcoxon signed-rank test.

Functional status improved significantly over time. The WHO functional class showed a marked shift toward lower severity (*W* = 99; *p* = 0.002; effect size *d* = 0.89; Figure 2). At baseline, most patients were classified as WHO functional class II–III (84%); by 24 weeks, the majority had improved to class I–II (80%; *p* = 0.002).

### 3.2. Changes in Risk Stratification at 24 Weeks

**Figure 3:**
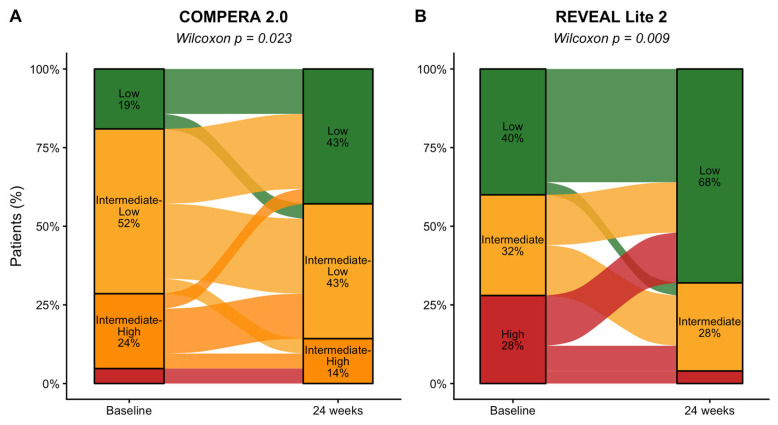
Transitions in risk stratification (baseline and 24-weeks) according to (**A**) COMPERA 2.0 and (**B**) REVEAL Lite 2. *p* values correspond to the Wilcoxon signed-rank test.

Risk stratification outcomes are presented in Figure 3. According to the COMPERA 2.0 model (Figure 3A), the proportion of patients classified as low risk increased from 19% at baseline to 43% at 24 weeks. Concurrently, the proportion of patients in higher-risk categories decreased, with no patients remaining in the high-risk category at follow-up.

Similarly, REVEAL Lite 2 (Figure 3B) demonstrated a shift toward lower-risk categories. The proportion of low-risk patients increased from 40% to 68%, while the proportion of high-risk patients decreased from 28% to 4%. These changes were statistically significant for both scoring systems (COMPERA 2.0: *p* = 0.023; REVEAL Lite 2: *p* = 0.009).

### 3.3. Agreement Between COMPERA 2.0 and REVEAL Lite 2

**Figure 4:**
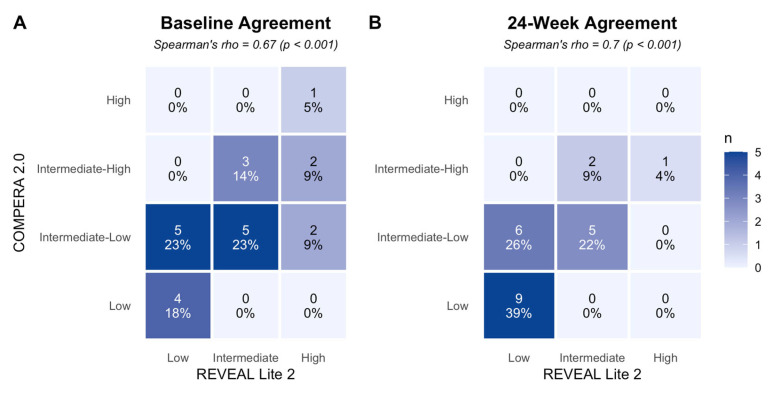
Concordance between COMPERA 2.0 and REVEAL Lite 2 risk classifications at (**A**) baseline and (**B**) at 24 weeks. Spearman correlation coefficients (*ρ*) and corresponding *p* values are shown in each panel.

Agreement between COMPERA 2.0 and REVEAL Lite 2 risk classifications was assessed at baseline and at 24 weeks (Figure 4). At follow-up, concordance between the two tools was strong (Spearman’s *ρ* = 0.70; *p* < 0.001). Baseline agreement was similarly high (*ρ* = 0.67; *p* < 0.001), indicating consistent risk classification across both time points.

### 3.4. Stratified Analysis According to Eisenmenger Syndrome

Stratified analyses based on the presence of Eisenmenger syndrome are presented in Table 2. The ART mixed model confirmed a statistically significant main effect of time for WHO functional class (F = 11.55; *p* = 0.002) and REVEAL Lite 2 risk strata (F = 11.03; *p* = 0.003), demonstrating robust clinical improvement. Crucially, no statistically significant time-by-group interactions were observed for any of the clinical or risk variables.

## 4. Discussion

This real-world study provides evidence on the potential benefits of sequential add-on therapy in patients with Group 1.4 PAH. The main findings are as follows: (a) a significant shift toward lower-risk categories after 24 weeks of ERA + PDE5i therapy; (b) improved functional status, as reflected by WHO functional class; (c) a significant reduction in BNP levels, supporting its role as a marker of therapeutic response; and (d) consistent findings in a Latin American real-world cohort, including patients with Eisenmenger syndrome.

Risk stratification plays a vital role in determining clinical management and predicting outcomes in patients with PAH. Over the past five years, the field has transitioned from broad assessments to highly refined multi-strata algorithms that separate patients with greater granular precision towards precision medicine. At baseline, a comprehensive three-strata model remains recommended to guide initial dual or triple combination therapies. During follow-up, a simplified four-strata model is preferred [15]. Our results reinforce the usefulness of both strategies in the initial evaluation and follow-up of patients with Group 1.4 pulmonary arterial hypertension; the decision to use either of the two tools will be made by the clinician in the context of daily patient care to determine the best treatment strategy and subsequent medication adjustments.

In this regard, contemporary PAH management has shifted from a static diagnostic approach to a dynamic, goal-directed strategy aimed at achieving and maintaining a low-risk profile which has been consistently associated with improved survival [6,16]. Although early trials focused on monotherapy, subsequent evidence showed that treatment goals were rarely achieved and that disease progression remained frequent [17]. The SERAPHIN trial, which included a subgroup of patients with Group 1.4 PAH, demonstrated that macitentan significantly reduced morbidity and mortality compared with placebo in PAH [18]. Similarly, Pulido et al. [19] reported reduced major clinical events with ERA therapy, confirming the clinical benefit of sustained pathway inhibition.

Even though these studies primarily included patients with idiopathic PAH, they established that early modification of pulmonary vascular remodeling improves clinical outcomes. Consistent with this, systematic reviews and meta-analyses support early combination therapy either as upfront or sequential add-on therapy. In particular, the combination of ERA and PDE5i has consistently shown superiority over monotherapy in low- and intermediate-risk patients, with higher rates of achieving low-risk status even after adjustment for comorbidities [20,21].

Current medical evidence highlights that while a broad multiparametric three-strata model establishes a secure baseline profile, using a simplified four-strata noninvasive score during subsequent follow-ups offers the most optimal balance of high prognostic accuracy and clinical practicality [15].

Our findings extend this evidence to Group 1.4 PAH, a population underrepresented in clinical trials. We observed a clear shift toward lower mortality risk at 24 weeks, independent of Eisenmenger syndrome status and consistent across COMPERA 2.0 and REVEAL Lite 2.0. The improvement in WHO functional class further supports a clinically meaningful treatment effect, given its established prognostic relevance in PAH. These results suggest that combination therapy benefits may extend beyond idiopathic PAH to more complex phenotypes where a profound and rapid remodeling of cardiopulmonary hemodynamics might occur. Recent clinical data has shown that, as soon as 4 weeks from initiation, sequential add-on therapy ensures that patients do not experience the therapeutic inertia that historically allowed the pulmonary vasculature to irreversibly deteriorate, with greater impact in pulmonary vascular resistance, attenuation of mean pulmonary artery pressure, and improvements in cardiac index and stroke volume index [22]. Nevertheless, the lack of continuous access to medication does not allow us to investigate a longer follow-up to strengthen the long-term clinical benefits.

Biomarker analysis supports these findings. BNP levels decreased significantly in our cohort, reflecting reduced right ventricular wall stress. BNP and NT-proBNP are among the most robust prognostic markers in PAH, and serial assessment is recommended for monitoring treatment response and residual risk [23]. The parallel improvement in BNP and risk stratification reinforces the internal consistency and biological plausibility of our results.

In contrast, no significant change was observed in 6MWD at 24 weeks. Although widely used, the 6MWD test has limited sensitivity to detect meaningful changes in small cohorts or in patients with advanced structural disease [24]. It is also influenced by musculoskeletal and non-cardiopulmonary factors, contributing to substantial intraindividual variability [25]. These factors likely explain the discrepancy between improvements in risk stratification and biomarkers versus functional walking distance. Nevertheless, the values observed are consistent with previous Latin American registry data [12].

Regionally, our findings align with the RESPHIRAR registry from southern Brazil, which reported a similar distribution of Group 1.4 PAH, including congenital heart disease (30.2%) and connective tissue disease (23.5%) [26]. Demographic patterns, including female predominance and diagnosis in the fourth decade of life, were also comparable across Latin American cohorts, contrasting with European and North American registries, where patients are typically older. The RESPHIRAR registry further confirmed the utility of REVEAL Lite 2.0 for risk stratification in real-world practice, supporting our findings.

Although comorbidities may influence disease trajectory and treatment response in PAH, combination therapy, either upfront or sequential add-on remains beneficial across heterogeneous patient groups [27]. In congenital heart disease and Eisenmenger syndrome, progressive right ventricular failure remains the main cause of morbidity and mortality [28], highlighting the importance of early intervention before irreversible cardiac dysfunction occurs.

Evidence in Eisenmenger syndrome remains limited. The MAESTRO trial evaluating macitentan did not demonstrate improvement in 6MWD at 16 weeks. However, consistently with our findings, improvements were observed in functional class, NT-proBNP, and pulmonary vascular resistance [29]. These data suggest that meaningful therapeutic benefit may be better captured by risk stratification and biomarker changes than by exercise capacity alone. In this context, our results support the ability of sequential add-on therapy to achieve favorable risk reclassification even in this complex phenotype.

Limitations: Two of the main drawbacks are the retrospective design and the absence of a comparison group, which limit the ability to attribute these changes exclusively to the treatment; however, all consecutively treated eligible patients were included. It is highly recommended that the findings of our single-center study involving a small group of patients be further validated in subsequent multicenter studies including a larger number of participants. Subgroup analyses (e.g., Eisenmenger syndrome) may have been underpowered to detect subtle differences or interactions. Hemodynamic follow-up was not systematically performed. However, this is consistent with routine clinical practice, where noninvasive risk assessment tools integrating clinical and biochemical variables are increasingly used for treatment monitoring and risk reassessment. Finally, although ERAs were approved by the Food and Drug Administration in 2013, they were not available at our institution until 2020 and were not incorporated into combination therapy (either upfront or sequential add-on) until January 2022. In addition, intermittent drug shortages limited consistent access to therapy, restricting continuous long-term treatment and limiting follow-up to 24 weeks. This is clinically relevant, as adequate treatment adherence (proportion of days covered ≥80%) has been associated with reduced hospitalization, slower disease progression, and lower mortality, whereas treatment interruption increases adverse clinical outcomes [30].

Due to the small size of our sample, the absence of significant differences in some stratified analyses might be related to a type II error. Larger studies are needed to identify other significant changes including mortality; however, it should be emphasized that, according to the reviewed literature, our study is the first in Latin America to include patients with Group 1.4 pulmonary hypertension receiving sequential add-on therapy.

## 5. Conclusions

In this real-world cohort from Mexico, ERA + PDE5i sequential add-on therapy was associated with a significant shift toward lower-risk strata in patients with Group 1.4 PAH. Improvements in COMPERA 2.0 and REVEAL Lite 2 scores, together with significant reductions in BNP levels and improved functional status, support the utility of dynamic risk stratification as a therapeutic target. These findings provide regional evidence in a subgroup underrepresented in clinical trials and suggest that sequential add-on therapy may favorably modify the prognostic profile even in complex PAH phenotypes. Larger studies with longer follow-up are needed to confirm its impact on long-term outcomes.

## Figures and Tables

**Table 1:** Demographics and clinical characteristics (*n* = 25).

Variable	Median (IQR)/*n* (%)
Age (yr)	37 (31–49)
Female sex	24 (96%)
BMI (kg/m^2^)	28.1 (25.8–31.1)
Pulmonary hypertensive subgroup	
1.4.1 Connective tissue disease	7 (24%)
1.4.4 Congenital heart disease	18 (72%)
Complex congenital heart disease	6 (33%)
Eisenmenger syndrome	16 (64%)

BMI, body mass index. Data are presented as median (interquartile range) or frequency (percentage).

**Table 2:** Mixed ANOVA results for clinical and risk variables, controlling for Eisenmenger syndrome.

Clinical Variable	Within-Subject Effect (Time)		Between-Subject Effect (Group)		Time × Group Interaction	
	*F* ^a^	*p*	*F* ^a^	*p*	*F* ^a^	*p*
BNP (pg/mL)	1.42	0.245	1.29	0.268	0.52	0.478
6 min walk distance (m)	0.10	0.750	0.003	0.956	0.75	0.397
TAPSE/sPAP ratio	3.98	0.061	0.06	0.803	0.43	0.521
Tricuspid regurgitation jet velocity (m/s)	0.68	0.422	0.24	0.632	0.06	0.813
WHO functional class	11.55	0.002	0.06	0.809	0.30	0.592
COMPERA 2.0 risk strata	4.18	0.054	0.005	0.943	0.44	0.512
REVEAL Lite 2 risk strata	11.03	0.003	0.61	0.442	0.03	0.866

BNP, brain natriuretic peptide; TAPSE, tricuspid annular plane systolic excursion; sPAP, systolic pulmonary artery pressure; WHO, World Health Organization; COMPERA 2.0 and REVEAL Lite 2: risk stratification models in pulmonary arterial hypertension. Within-subject (time), between-subject (group), and time × group interaction effects are presented. ^a^ Analyses were performed using the Aligned Rank Transform (ART) procedure due to violation of the normality assumption in several clinical variables.

## Data Availability

The data presented in this study are available from the corresponding author upon reasonable request. The data are not publicly available due to privacy and ethical restrictions.

## Abbreviations

6MWD	6 min walk distance
BMI	Body mass index
BNP	Brain natriuretic peptide
COMPERA	Comparative, Prospective Registry of Newly Initiated Therapies for Pulmonary Hypertension
ERA	Endothelin receptor antagonist
ESC	European Society of Cardiology
ERS	European Respiratory Society
IQR	Interquartile range
mMRC	Modified Medical Research Council
NT-proBNP	N-terminal pro B-type natriuretic peptide
PAH	Pulmonary arterial hypertension
PAH-CHD	Pulmonary arterial hypertension associated with congenital heart disease
PAH-CTD	Pulmonary arterial hypertension associated with connective tissue disease
PDE5i	Phosphodiesterase type 5 inhibitor
REVEAL	Registry to Evaluate Early and Long-term PAH Disease Management
STROBE	Strengthening the Reporting of Observational Studies in Epidemiology
TAPSE	Tricuspid annular plane systolic excursion
TTE	Transthoracic echocardiography
WHO	World Health Organization
yr	Year

## References

[1] Humbert M., Kovacs G., Hoeper M.M., Badagliacca R., Berger R.M.F., Brida M., Carlsen J., Coats A.J.S., Escribano-Subias P., Ferrari P. (2023). 2022 ESC/ERS guidelines for the diagnosis and treatment of pulmonary hypertension. Eur. Respir. J..

[2] Kovacs G., Bartolome S., Denton C.P., Gatzoulis M.A., Gu S., Khanna D., Badesch D., Montani D. (2024). Definition, classification and diagnosis of pulmonary hypertension. Eur. Respir. J..

[3] Arvanitaki A., Gatzoulis M.A., Opotowsky A.R., Khairy P., Dimopoulos K., Diller G.P., Giannakoulas G., Brida M., Griselli M., Grünig E. (2022). Eisenmenger syndrome: *JACC* state-of-the-art review. J. Am. Coll. Cardiol..

[4] Jone P.N., Ivy D.D., Hauck A., Karamlou T., Truong U., Coleman R.D., Sandoval J.P., Marín M.J.d.C., Eghtesady P., Tillman K. (2023). Pulmonary hypertension in congenital heart disease: A scientific statement from the American Heart Association. Circ. Heart Fail..

[5] Boucly A., Weatherald J., Savale L., Jaïs X., Cottin V., Prevot G., Picard F., de Groote P., Jevnikar M., Bergot E. (2017). Risk assessment, prognosis and guideline implementation in pulmonary arterial hypertension. Eur. Respir. J..

[6] Hoeper M.M., Pausch C., Olsson K.M., Huscher D., Pittrow D., Grünig E., Staehler G., Vizza C.D., Gall H., Distler O. (2022). COMPERA 2.0: A refined four-stratum risk assessment model for pulmonary arterial hypertension. Eur. Respir. J..

[7] Benza R.L., Kanwar M.K., Raina A., Scott J.V., Zhao C.L., Selej M., Elliott C.G., Farber H.W. (2021). Development and validation of an abridged version of the REVEAL 2.0 risk score calculator, REVEAL Lite 2. Chest.

[8] Galiè N., Barberà J.A., Frost A.E., Ghofrani H.A., Hoeper M.M., McLaughlin V.V., Peacock A.J., Simonneau G., Vachiery J.-L., Grünig E. (2015). Initial use of ambrisentan plus tadalafil in pulmonary arterial hypertension. N. Engl. J. Med..

[9] Muller A., Escribano-Subias P., Fernandes C.C., Fontana M., Lange T.J., Söderberg S., Gaine S. (2024). Real-world management of patients with pulmonary arterial hypertension: Insights from EXPOSURE. Adv. Ther..

[10] Orozco-Levi M., Souza R., Bluro I.M., Harley J., Hernández Oropeza J.L., Lescano A., Meyer G., Pineda T., Ramirez A., Small M. (2024). Pathway to care, treatment and disease burden of pulmonary arterial hypertension: A real-world survey of physicians and patients in Latin America. BMJ Open.

[11] Valverde A.B., Soares J.M., Viana K.P., Gomes B., Soares C., Souza R. (2018). Pulmonary arterial hypertension in Latin America: Epidemiological data from local studies. BMC Pulm. Med..

[12] García-Aguilar H., Vázquez S.G., Trejo K.S., Juárez Y.E., Juárez Vásquez K.I., Sarmiento Sánchez E.S., Molina H.S. (2021). Healthcare resource utilization and costs in pediatric pulmonary arterial hypertension in a third-level hospital in Mexico. J. Comp. Eff. Res..

[13] Colón F., Gutiérrez A., García A.G., Jiménez L., Arbaje D.E., Zayas N. (2021). Pulmonary arterial hypertension and renal impairment in patients from the National Institute of Cardiology Dr. Ignacio Chávez, Mexico. Arch. Peru. Cardiol. Cir. Cardiovasc..

[14] Von Elm E., Altman D.G., Egger M., Pocock S.J., Gøtzsche P.C., Vandenbroucke J.P., for the STROBE Initiative (2007). The strengthening the reporting of observational studies in epidemiology (STROBE) statement: Guidelines for reporting observational studies. Lancet.

[15] Dardi F., Boucly A., Benza R., Frantz R., Mercurio V., Olschewski H., Rådegran G., Rubin L.J., Hoeper M.M. (2024). Risk stratification and treatment goals in pulmonary arterial hypertension. Eur. Respir. J..

[16] Boucly A., Montani D., Bauer F., Artaud-Macari E., Bergot E., Boissin C., Chaouat A., Cottin V., Dauphin C., Degano B. (2025). Initial therapy in patients with pulmonary arterial hypertension and cardiovascular comorbidities. Eur. Respir. J..

[17] Hassoun P.M. (2021). Pulmonary arterial hypertension. N. Engl. J. Med..

[18] Said K. (2014). Macitentan in pulmonary arterial hypertension: The SERAPHIN trial. Glob. Cardiol. Sci. Pract..

[19] Pulido T., Adzerikho I., Channick R.N., Delcroix M., Galiè N., Ghofrani H.A., Jansa P., Jing Z.-C., Le Brun F.-O., Mehta S. (2013). Macitentan and morbidity and mortality in pulmonary arterial hypertension. N. Engl. J. Med..

[20] Oba Y., Maduke T., Fakhouri E.W., Goite Y. (2025). Phosphodiesterase type 5 inhibitor plus endothelin receptor antagonist compared to either alone for pulmonary arterial hypertension. Cochrane Database Syst. Rev..

[21] McLaughlin V.V., Sitbon O., Chin K.M., Galiè N., Hoeper M.M., Kiely D.G., MacDonald G., Martin N., Mathai S.C., Peacock A. (2024). Initial combination therapy with macitentan and tadalafil in patients with pulmonary arterial hypertension, with and without Cardiac Comorbidities. Eur. J. Heart Fail..

[22] Kramer T., Nattmann P., Gerhardt F., Stafiej P., Dumitrescu D., Ten Freyhaus H., Wißmüller M., Hohmann C., Baldus S., Rosenkranz S. (2024). Impact of rapid sequential combination therapy on distinct haemodynamic measures in newly diagnosed pulmonary arterial hypertension. ESC Heart Fail..

[23] Lewis R.A., Durrington C., Condliffe R., Kiely D.G. (2020). BNP/NT-proBNP in pulmonary arterial hypertension. Eur. Respir. Rev..

[24] Harari S., Wells A.U., Wuyts W.A., Nathan S.D., Kirchgaessler K.U., Bengus M., Behr J. (2022). The 6-min walk test as a primary end-point in interstitial lung disease. Eur. Respir. Rev..

[25] Enjuanes C., Moliner-Borja P., Meroño O., Comín-Colet J. (2016). Limitaciones de la prueba de marcha de 6 minutos en insuficiencia cardiaca crónica. Rev. Esp. Cardiol..

[26] Spilimbergo F.B., Rodrigues R.P., Dias-Pinto M.C., Blanco D.C., Barbieri G.M., Andrade-Lima M., Leal Fagundes A., Gazzana M.B., Roncato G., Mello M.M. (2023). Risk assessment validation in pulmonary arterial hypertension: Data from the RESPHIRAR study. Pulm. Circ..

[27] Rosenzweig E.B., Krishnan U. (2021). Congenital heart disease-associated pulmonary hypertension. Clin. Chest Med..

[28] Provencher S., Mai V., Bonnet S. (2024). Managing pulmonary arterial hypertension with cardiopulmonary comorbidities. Chest.

[29] Gatzoulis M.A., Landzberg M., Beghetti M., Berger R.M., Efficace M., Gesang S., He J., Papadakis K., Pulido T., Galiè N. (2019). Evaluation of macitentan in patients with Eisenmenger syndrome. Circulation.

[30] De Marco T., Paoli C.J., Germack H.D., Croteau N.S., Simeone J.C., Tang F., Doad G., Panjabi S., Farber H. (2026). Relationship between adherence and outcomes in pulmonary arterial hypertension. Respir. Med..

